# Severe COVID-19 Recovery Is Associated with Timely Acquisition of a Myeloid Cell Immune-Regulatory Phenotype

**DOI:** 10.3389/fimmu.2021.691725

**Published:** 2021-06-23

**Authors:** Amelia C. Trombetta, Guilherme B. Farias, André M. C. Gomes, Ana Godinho-Santos, Pedro Rosmaninho, Carolina M. Conceição, Joel Laia, Diana F. Santos, Afonso R. M. Almeida, Catarina Mota, Andreia Gomes, Marta Serrano, Marc Veldhoen, Ana E. Sousa, Susana M. Fernandes

**Affiliations:** ^1^ Instituto de Medicina Molecular João Lobo Antunes, Faculdade de Medicina, Universidade de Lisboa, Lisbon, Portugal; ^2^ Clinica Universitária de Medicina Intensiva, Faculdade de Medicina, Universidade de Lisboa, Lisbon, Portugal; ^3^ Serviço de Medicina II, Hospital de Santa Maria, Centro Hospitalar Universitário Lisboa Norte, Lisbon, Portugal; ^4^ Serviço de Medicina Intensiva, Hospital de Santa Maria, Centro Hospitalar Universitário Lisboa Norte, Lisbon, Portugal

**Keywords:** innate immunity, SARS-CoV-2, COVID-19, M2-like differentiation, immune-regulation

## Abstract

After more than one year since the COVID-19 outbreak, patients with severe disease still constitute the bottleneck of the pandemic management. Aberrant inflammatory responses, ranging from cytokine storm to immune-suppression, were described in COVID-19 and no treatment was demonstrated to change the prognosis significantly. Therefore, there is an urgent need for understanding the underlying pathogenic mechanisms to guide therapeutic interventions. This study was designed to assess myeloid cell activation and phenotype leading to recovery in patients surviving severe COVID-19. We evaluated longitudinally patients with COVID-19 related respiratory insufficiency, stratified according to the need of intensive care unit admission (ICU, n = 11, and No-ICU, n = 9), and age and sex matched healthy controls (HCs, n = 11), by flow cytometry and a wide array of serum inflammatory/immune-regulatory mediators. All patients featured systemic immune-regulatory myeloid cell phenotype as assessed by both unsupervised and supervised analysis of circulating monocyte and dendritic cell subsets. Specifically, we observed a reduction of CD14lowCD16+ monocytes, and reduced expression of CD80, CD86, and Slan. Moreover, mDCs, pDCs, and basophils were significantly reduced, in comparison to healthy subjects. Contemporaneously, both monocytes and DCs showed increased expression of CD163, CD204, CD206, and PD-L1 immune-regulatory markers. The expansion of M2-like monocytes was significantly higher at admission in patients featuring detectable SARS-CoV-2 plasma viral load and it was positively correlated with the levels of specific antibodies. In No-ICU patients, we observed a peak of the alterations at admission and a progressive regression to a phenotype similar to HCs at discharge. Interestingly, in ICU patients, the expression of immuno-suppressive markers progressively increased until discharge. Notably, an increase of M2-like HLA-DRhighPD-L1+ cells in CD14++CD16− monocytes and in dendritic cell subsets was observed at ICU discharge. Furthermore, IFN-γ and IL-12p40 showed a decline over time in ICU patients, while high values of IL1RA and IL-10 were maintained. In conclusion, these results support that timely acquisition of a myeloid cell immune-regulatory phenotype might contribute to recovery in severe systemic SARS-CoV-2 infection and suggest that therapeutic agents favoring an innate immune system regulatory shift may represent the best strategy to be implemented at this stage.

## Introduction

After more than 1 year from the coronavirus disease (COVID-19) outbreak in Wuhan, China, more than 100 million cases and 2,5 million deaths have been confirmed worldwide (1). The etiological agent, the severe acute respiratory syndrome Coronavirus (SARS-CoV)-2, determines a mild illness in the majority of the patients. In 5% of the cases, rapid viral replication, immune cell infiltration, and uncontrolled inflammatory response occur, causing acute lung injury (ALI) or acute respiratory distress syndrome (ARDS), with or without multi-organ failure, resulting in a high case-fatality ratio (2).

Critically ill COVID-19 patients represent the bottleneck of the pandemic management, leading to an overwhelming impact on available health care resources. Most of the available therapies used for severe cases are still today of supportive nature, while several immune-modulating agents are under trial (3, 4). Cytokine storm and macrophage activation syndrome were reported in fatal COVID-19 cases. Interferon-γ (IFN-γ), interleukin-1 (IL-1), IL-6, tumor necrosis factor-α (TNF-α), and IL-18 have central immunopathogenic roles in the hyper-inflammation (5, 6). Decoy receptors for pro-inflammatory cytokines such as IL-1RA, as well as anti-inflammatory cytokines such as IL-10, constitute negative feedback mechanisms preventing immune hyper-activation and immunopathology (5).

Interestingly, Coronaviruses (CoVs) encode multiple proteins that antagonize the activation of type I, II, and III IFN responses (7–12). The inhibition of IFN pathways has been associated to the insidious clinical course of COVID-19, until late deterioration (7). Several reports have assessed aspects of systemic innate immune response to SARS-CoV-2, initially with contrasting results: increased or decreased levels of classical, intermediate, and non-classical monocytes and presence of both pro- and anti-inflammatory markers in circulating myeloid cells have been described (8, 9). More recently, systemic loss and functional impairment of pro-inflammatory monocytes, conventional and plasmacytoid dendritic cells (pDCs) populations, sustaining for a loss of M1-like/pro-inflammatory cells, were reported as distinctive features of the severe compared to moderate disease (10–12).

Nevertheless, to our knowledge, few data are available on longitudinal comprehensive characterization of myeloid cell phenotype, allowing elucidation of their role in COVID-19 recovery.

Here we performed a detailed longitudinal evaluation of circulating monocytes/macrophages and dendritic cells (DCs), along with a wide range of circulating cytokines and chemokines. We aimed to clarify if these elements of the innate immune response might direct towards inflammation or immune-suppression in COVID-19 associated with severe symptoms. Moreover, we assessed in hospitalized patients whether intensive care unit (ICU) requirement or symptoms resolution and discharge might be linked to a particular systemic myeloid cell and circulating cytokine/chemokine signature, with the ultimate goal to identify pathways to be targeted to induce the recovery.

## Materials and Methods

### Patients and Healthy Controls

Twenty-one adult patients affected by COVID-19 related pneumonia, admitted at the Centro Hospitalar Universitário Lisboa Norte (CHULN, Lisboa, Portugal), between April and October 2020, and 11 healthy controls (HCs), were enrolled in the study (**Table 1**). Age and sex distribution were homogeneous in patient and healthy control groups. Informed consent was obtained from all participants and the study was approved by the Ethics committee at the CHULN/Faculdade de Medicina da Universidade de Lisboa. SARS-CoV-2 infection was confirmed by real-time PCR (RT-PCR) for nucleic acid testing of nasopharyngeal swabs.

**Table 1:** Clinical and routine laboratory data from patients and healthy controls.

Clinical variables		No-ICU	ICU	HCs	p (Global)^a^	p (ICU *vs* HCs)^b^	p (No ICU *vs* HCs)^b^	p (ICU *vs* No-ICU)^b^
n		9	11	11				
Age in years		50 (39–65)	57 (45.5–64)	58 (39–65)	0.485	0.965	0.965	0.965
Male sex, n (%)^c^		7 (77.7)	10 (91)	9 (73)	0.671	0.315	0.882	0.413
Arterial hypertension, n (%)^c^		4 (44)	5 (46)	1 (9)	0.264	0.056	0.069	0.964
Diabetes type 2, n (%)^c^		3 (33)	3 (27)	0	0.251	0.062	**0.038**	0.2942
Obesity, n (%)^c^		1 (11)	5 (45)	0	**0.032**	**0.011**	0.257	0.095
Lung emphysema, n (%)^c^		0	2 (18)	0	0.313	0.138	>0.999	0.178
Time from symptoms start to admission (days)		8 (4–10)	9 (7–12)	NA	0.302	NA	NA	0.302
Time from symptoms start to recovery (days)		12 (11–17)	20 (17–21.5)	NA	**0.009**	NA	NA	**0.009**
Time from admission to discharge (days)		9 (8–10)	12 (9.5–15)	NA	**0.034**	NA	NA	**0.034**
P/F	A	287.4 (270–323)	122.1 (104.5–272.2)	NA	NA	NA	NA	**<0.001**
	D	447.6 (340–461)	283.3 (273–392.9)	NA	NA	NA	NA	**0.03**
CRP (mg/dl)	A	5.44 (2.26–7.03)	23.7 (12.5–26.5)	NA	NA	NA	NA	**0.005**
PCT (ng/ml)	A	0.14 (0.11–0.41)	0.21 (0.15–0.83)	NA	NA	NA	NA	0.440
Ferritin (mg/dl)	A	949 (344–1,374)	1,030 (435.3–1,998)	NA	NA	NA	NA	0.450
D-dimers (ng/ml)	A	0.43 (0.19–53)	0.23 (0.18–0.63)	NA	NA	NA	NA	0.712
LDH (U/L)	A	366 (222–417.5)	372 (293–393)	NA	NA	NA	NA	0.736
Lymphocytes/µl	A	1,390 (835–2,108)	870 (840–1,160)	1,940 (1,423–2,200)	**0.005**	**0.003**	0.152	0.146
	D	1,810 (1,675–1,995)	1,982 (1,269–2,680)		0.8464	0.710	0.661	0.898
Neutrophils/µl	A	3,923 (2,385–4,952)	7,447 (4,687–11,793)	3,228 (2,521–6,390)	**0.011**	**0.010**	0.924	**0.007**
	D	3,349 (2,792–4,891)	5,557 (4,303–9,620)		**0.067**	**0.045**	0.978	**0.**060
Lymphocytes/neutrophils	A	0.316 (0.26–0.65)	0.146 (0.086–0.380)	0.509 (0.47–0.605)	**0.013**	**0.008**	0.194	**0.038**
	D	0.588 (0.35–0.69)	0.264 (0.142–0.559)		0.098	**0.005**	0.892	**0.032**
Monocytes/µl	A	321.9 (228–503)	376.8 (210.6–658.5)	398 (274.8–732.8)	0.872	0.833	0.640	0.766
	D	397 (347–595)	636 (472–1,057)		0.099	0.055	0.890	0.112
Basophils/µl	A	15.09 (6.35–31.77)	31.2 (16.9–42.32)	31.6 (16.4–63)	0.150	0.550	0.077	0.152
	D	19.56 (10.1–26.8)	30.9 (15.86–54.2)		0.273	0.589	0.109	0.364
Eosinophils/µl	A	12.5 (5.5–40.88)	24.39 (6.85–60.18)	114.8 (96.48–297)	**<0.001**	**<0.001**	**<0.001**	0.602
	D	116 (36.95–142.7)	42 (23.21–58.41)		**0.008**	**<0.001**	0.364	0.190
Detectable SARS-Cov-2 Plasma viral Load, n (%)^c^	A	3 (33%)	10 (91%)	NA	**0.007**	NA	NA	**0.007**
	D	0 (0%)	0 (0%)					
SARS-Cov-2 Plasma viral Load in patients with detectable levels (cps/ml)	A	111.7 (33.91–563.5)	131 (36.1–713)	NA	NA	NA	NA	0.864
	D	NA	NA	NA	NA	NA	NA	NA
Treatment^c^:				NA		NA	NA	
Dexamethasone, n (%)		1 (11)	4 (36)		0.293			0.293
Other glucocorticoids, n (%)		1 (11)	5 (45)		0.619			0.619
Tocilizumab, n (%)		0 (0)	3 (27)		0.507			0.507
Lopinavir/Ritonavir, n (%)		6 (68)	2 (18)		0.123			0.123
Remdesivir, n (%)		2 (22)	4 (36)		0.632			0.632

Values expressed as medians (interquartile range) unless otherwise specified. P/F, Ratio of the partial pressure of arterial oxygen to the fraction of inspired oxygen; CRP, C reactive protein; PCT, procalcitonin; Ferritin: A, Admission; D, Discharge; NA, Not Applicable. Comparisons were done using ^a^One way ANOVA unless otherwise stated; ^b^Mann–Whitney U-test unless otherwise stated; ^c^Chi-squared test. Significant p values were shown in bold.

The first time point for clinical and laboratory evaluation was performed at admission to the intensive care unit (ICU group) and at hospital admission (HA) for the patients not requiring high flux nasal oxygen (HFNO patients) or mechanical ventilation (MV) respiratory support (No-ICU group). Afterwards, all patients were evaluated at recovery, when discharged from hospital or from ICU. Collection of all clinical information (**Table 1**) was monitored by the same clinician, that integrates the research team (SMF). At each time point, clinical data, whole blood, plasma, and serum were collected for all individuals. To obtain a more homogenous set of patient samples, the study participants were screened for co-infections, and one case of HIV-1/SARS-CoV-2 co-infection was excluded and considered to be analyzed separately. Furthermore, three No-ICU patients were lost at the follow up and two ICU patients died after the ICU admission time point. Whole blood was processed immediately after sampling and no difference in sample handling or material used existed among patient and control groups.

### Flow Cytometry

Multi-parameter flow cytometry for immune-phenotyping of circulating monocyte/macrophage and DCs was performed on whole blood, immediately after sampling.

In this and other studies we confirmed that if rapid sample processing was performed in whole blood no significant amount of dead cells are reported, therefore a live/dead marker was not used for this evaluation. After erythrocyte bulk lysis, 10 million leukocytes were incubated with a panel of fluorochrome-labelled antibodies, for 30 min at room temperature. The cell populations were stained with the following antibodies: anti-Slan Ef450, anti-CD141 BV510, anti-CD45 BV605, anti-HLA-DR BV650, anti-CD86 BV711, anti-PD-L1 BV785, anti-CD3 FITC, anti-CD19 FITC, anti-CD66b FITC, anti-CD14 PerCP-Cy5.5, anti-CD80 PE, anti-CD163 PE-CF594, anti-CD206 PE-Cy5, anti-CD123 PE-Cy7, anti-CD204 APC, anti-CD16 AF700, anti-CD1c APC-Cy7 (**Supplementary Table 1**). After fixation, cells were resuspended in PBS and acquired in a Fortessa X20 flow cytometer. The data were analyzed with FlowJo software (Version 10.7; Tree Star, Inc., Ashland, OR, USA).

For flow cytometry data analysis, both supervised and unsupervised approaches were implemented. Traditional manual hierarchical gating was applied on 2D scatterplots starting on a large lymphocyte/monocyte including gate (**Supplementary Figure 1**). After cell debris and doublets exclusion, based on forward and side scatter, monocytes, macrophages, and DCs were defined by selection of CD45 antigen-expressing cells, negative for the lineage markers CD66b, CD19, and CD3 (CD45+Lin− cells). CD56 was not used among lineage markers in order to prevent the exclusion of possible myeloid cells expressing CD56 (13).

For the unsupervised analysis, CD45+Lin− cells were gated and dimensionality reduction was applied through the t-distributed Stochastic Neighbour Embedding (tSNE) in Flow-Jo version 10.7. on 79,688 events from each patient and HC. Firstly, to obtain the cluster number definition, both X-Shift and Phenograph were used on the same datasets, evaluating the best clustering resolution visualized on the t-SNE images. The Phenograph clustering was excluded because of the higher number of clusters defined, several of which representing single outliers. The X-Shift cluster definition of 16 clusters in CD45+Lin− concatenated events was applied (14). Afterwards, to confirm and visualize the results on a minimal spanning tree, the dataset was re-clustered using FlowSOM (15).

Bidimensional hierarchical gating strategy was also used to further define in a CD45+Lin− gate the plasmocytoid dendritic cell (pDC), and the CD141+ and CD1c+ myeloid dendritic cell (mDC) subpopulations.

Within the CD14 positive cells, in the CD45+Lin− population, a trapezoidal gating strategy was applied uniformly to all patients and control samples to define the monocyte subsets as CD14++CD16− classical, CD14++CD16+ intermediate, CD14lowCD16+ non-classical (16, 17). Slan was used for non-classical monocyte sub-setting.

In monocyte and DC cell populations, the levels of expression of CD163, CD204, and CD206 (for M2-like polarization), PD-L1 (immune-regulation/suppression), HLA-DR, CD80, CD86 (activation, antigen presentation, and M1-like phenotype), were assessed.

For each studied individual unstained cells were used as control for gating of the negative and positive populations.

For each patient and control a hemogram with complete white blood cell count was performed from the same blood sampling at Santa Maria Hospital clinical laboratory. Absolute numbers of monocyte subsets were calculated by multiplying their percentual representation by the absolute monocyte count obtained at the clinical laboratory.

### Serum Proteins

Multiplex ELISA for 71 cytokines and chemokines was performed on the −80°C stored serum samples, from each time point, using a Multiplexing LASER Bead Assay (Human Cytokine Array/Chemokine Array 71-Plex Panel, HD71, Eve Technologies, Canada) while CCL28, RAGE, SP-D, IL-22BP were determined by Sandwich ELISA kits, as specified by the manufacturer (RayBiotech, GA).

SARS-CoV-2 specific antibody responses were evaluated through quantitative tests for IgM, IgG, and IgA against spike protein or its receptor binding domain, as previously described (18).

### SARS-CoV-2 Plasma Viral Load

Total RNA was extracted from collected plasma samples (560 µl) using QIAamp^®^ Viral RNA Mini Kit (QIAGEN), according to manufacturer’s instructions. SARS-CoV-2 viremia was quantified using the commercial RT-PCR amplification kit Bio-Rad SARS-CoV-2 ddPCR Test Kit (Bio-Rad) on QX200™ Droplet Digital PCR System (Bio-Rad), following manufacturer’s instructions. Each 20 μl ddPCR reaction used 5 μl of extracted RNA with samples in duplicate to quantify copies/reaction. Plasma samples with one of the two N1 or N2 regions or both regions detected were considered as positive samples and results were analyzed on QuantaSoft Analysis Pro (1.0.596). SARS-CoV-2 RNA concentrations (cp/ml) were finally calculated considering the extracted volume of plasma.

### Statistical Analysis

The statistical analysis was performed within and between different patient groups and in comparison to HCs. The Kruskal-Wallis and the Dunn’s multiple comparison tests were used to compare variables with continuous distribution in more than two groups. Wilcoxon matched-pairs signed rank test and Mann–Whitney U-test were used for paired and unpaired analyses of continuous data, respectively. Spearman’s Delta was done for hypothesis testing of correlations. Principal Component Analysis was performed for dimensionality reduction and evaluation of relevant analytes contributing to data variation, afterwards hierarchical clustering was applied. Ultimately, volcano plots were employed to quantify, in terms of fold changes and statistical significance, the most meaningful modifications in the variables analyzed. All values were presented as median (25^th^–75^th^ percentiles). Data were analyzed with R version 4.0.2., using the packages *heatmaply, EnhancedVolcano*, and *ggplot2* for data visualization and GraphPad Prism version 8 (GraphPad Software, San Diego, CA, USA). A p value <0.05 was considered statistically significant.

## Results

In order to investigate myeloid cell phenotype contributing to the recovery of patients with severe COVID-19, we compared the data obtained at admission and at discharge from the respiratory isolation units or the ICU, within patient group or between patient groups and HCs (**Table 1**). As expected, patients requiring ICU featured significantly longer hospital stay and lower ratio of the partial pressure of arterial oxygen to the fraction of inspired oxygen (P/f), both at admission and at discharge (**Table 1**). The use of steroids and other therapies with possible impact on the viral or immune response was comparable between the two patient groups (**Table 1**). Interestingly, SARS-CoV-2 was detectable in the plasma of all ICU patients at admission, but not at discharge. Although ICU patients had higher neutrophil counts and C reactive protein (CRP) serum levels, no difference was reported for procalcitonin (PCT), ferritin, and D-dimers in the two groups (**Table 1**).

Notably, both patient groups featured no significant changes in circulating monocyte counts (**Table 1**).

**Figure 1:**
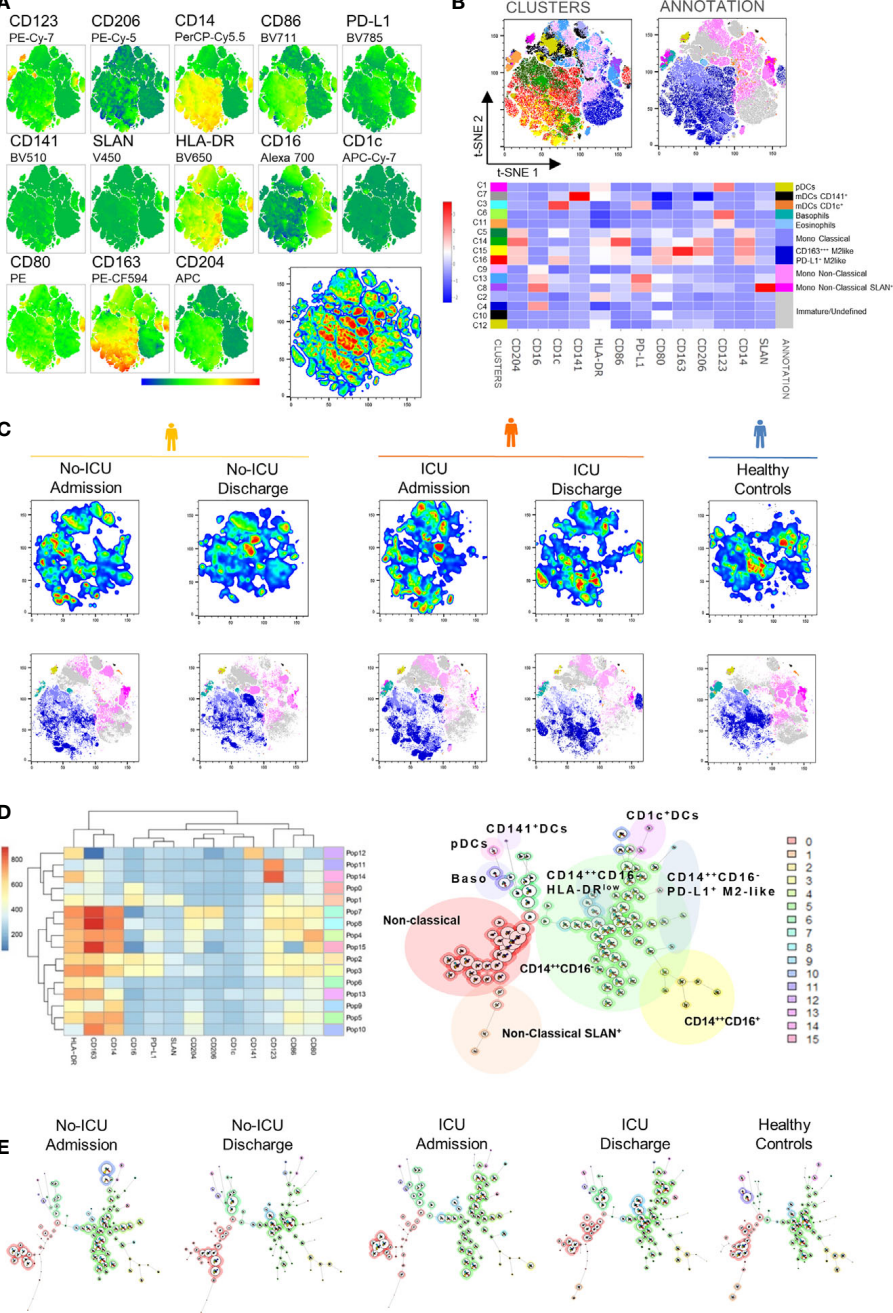
Unsupervised analysis of myeloid cells in COVID-19 patients and healthy controls. **(A)** Dimensionality reduction performed by t-distributed Stochastic Neighbor Embedding (t-SNE) on concatenated CD45+Lin− cells from both time-points and from patient and control groups. Bi-dimensional plots of concatenated samples showing marker distribution. **(B)** Unsupervised clustering performed using X-shift. Bi-dimensional plots showing the 16 clusters obtained (left) and the manual annotation of the clusters (right). Heatmap showing the marker expression in the 16 X-shift clusters. **(C)** Cluster distribution in patients at admission/discharge and in healthy controls. Bi-dimensional plots showing event density using pseudo-color (top) and cluster manual annotation (bottom). **(D)** Unsupervised clustering using FlowSOM. Heatmap showing relative marker expression (left) and self-organizing map with the obtained clusters (right). **(E)** FlowSOM cluster distribution in patients at admission/discharge and in healthy controls. Healthy controls (blue), No-ICU patients (yellow), and ICU patients (orange).

Monocytes, macrophages, and DCs were analyzed within the concatenated CD45+Lin− dataset (**Supplementary Figure 1**), through an unsupervised approach, starting with dimensionality reduction by tSNE (**Figure 1A**). Afterwards, X-Shift clustering was applied to obtain 16 different clusters (**Figures 1B, C**, **Supplementary Figure 2** and **Supplementary Table 2**). The CD45+Lin− dataset was then re-clustered with FlowSOM, to show the relative distribution of the populations in a self-organizing map defining a minimal spanning tree (**Figures 1D, E**).

Interestingly, all patients showed relevant changes in several cluster frequencies compared to HCs, but at different time points for No-ICU and ICU groups.

**Figure 2:**
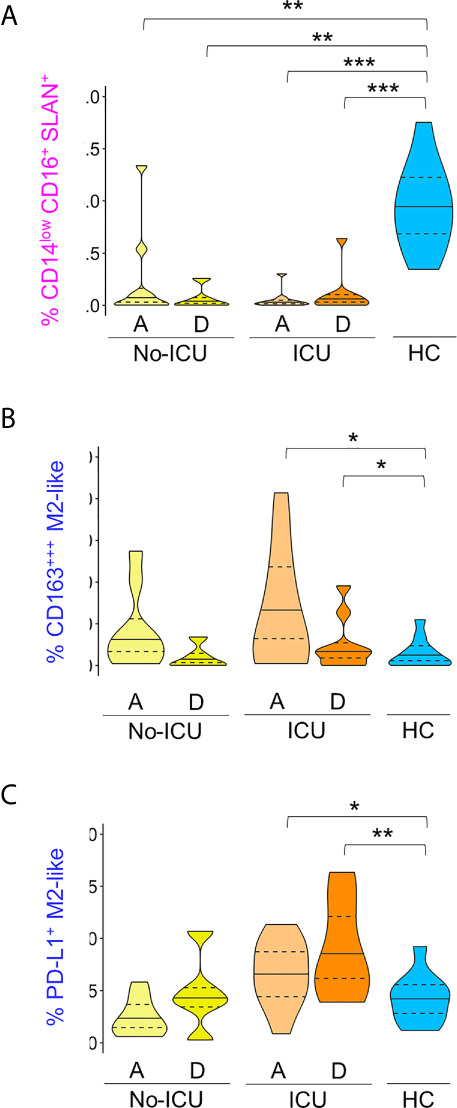
*M2-like and Slan+ monocyte clusters in COVID-19*. Frequency of clusters, manually annotated, identified using X-Shift within total CD45+Lin− cells: **(A)** In pink: CD14lowCD16+Slan+; **(B)** In dark blue: CD163+++M2-like; **(C)** In dark blue: PD-L1+M2-like. Comparisons were performed between patient groups and healthy controls using Mann–Whitney U-test. p values are shown as ***p < 0,001; **p < 0,01; *p < 0,05. No statistical differences were found between patient groups. ICU, Intensive care unit; A, admission; D, Discharge; HCs, Healthy controls.

The Cluster (C) 8, having the characteristics of non-classical monocytes expressing high levels of Slan, HLA-DR, PD-L1, CD80, and CD86, and low CD163, was significantly and persistently reduced in both patient groups until discharge, in comparison to healthy subjects (**Figures 1B, C**, **Figure 2A**, **Supplementary Figure 2** and **Supplementary Table 2**). In the spanning tree, a complete shrinking of the Slan+ CD14lowCD16+ nodes was observed in No-ICU patients at HA, only partially re-appearing in the discharge time-point map. A reduction of this branch was also present in the ICU group, but more notably at discharge (**Figure 1E**).

On the other hand, the C15, with a classical monocyte phenotype, characterized by the highest CD163 levels, low/intermediate HLA-DR, CD86, and CD204, and low CD80 (CD163+++M2-like monocytes), was significantly expanded, especially in ICU patients at admission and discharge (**Figures 1B, C**, **Figure 2B**, **Supplementary Figure 2** and **Supplementary Table 2**).

Importantly, C16, a classical monocyte population with positivity for M2 markers, high HLA-DR, and PD-L1 levels (PD-L1+M2-like monocytes), showed the highest percentages in ICU patients at discharge (**Figures 1B, C**, **Figure 2C** **Supplementary Figure 2** and **Supplementary Table 2**).

In the FlowSOM maps, classical monocytes with higher expression of scavenger receptors, HLA-DR and PD-L1, as well as the HLA-DRlow classical monocytes, were mainly observable at discharge in ICU patients (**Figures 1D, E**).

No differences were found for less differentiated CD14++CD16− monocytes (C5 and C14), as well as for other CD14lowCD16+ clusters (C9 and C13) (**Figures 1B, C**, **Supplementary Figure 2** and **Supplementary Table 2**).

**Figure 3:**
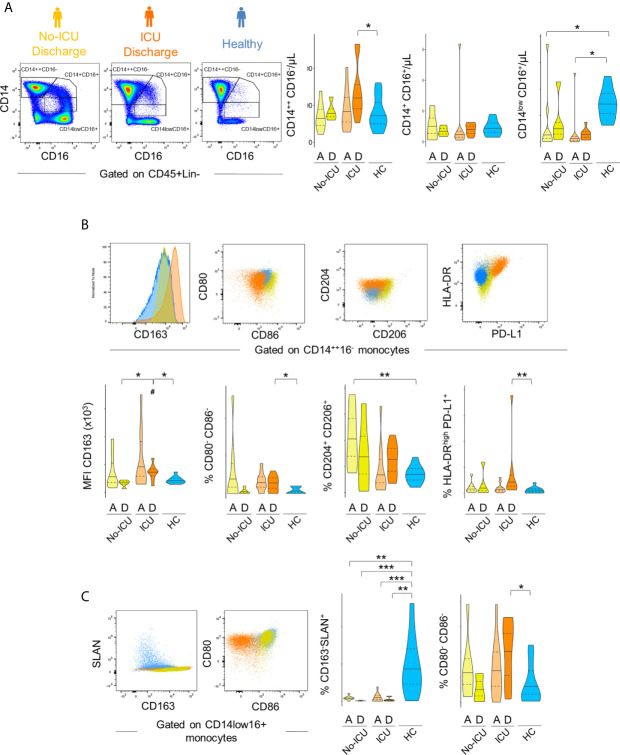
Immune regulatory phenotype of monocytes in severe COVID-19 assessed by bi-dimensional hierarchical gating strategy. **(A–C)** Illustrative dot plots of the analysis performed in a representative No-ICU patient at discharge (yellow) and in an ICU patient at discharge (orange), as well as in a healthy control (blue) are shown; **(A)** Violin graphs show absolute counts of the main monocyte subsets; **(B)** CD163 MFI and proportion of CD80^−^CD86^−^, CD204^+^CD206^+^, and HLA-DR^high^PD-L1^+^ subsets within classical (CD14^++^CD16^−^) monocytes; **(C)** Proportion of CD163^−^Slan^+^ and CD80^−^CD86^−^ within non-classical (CD14^low^CD16^+^) monocytes. There were no significant differences between admission and discharge in both ICU and No-ICU patient groups (Wilcoxon matched-pairs signed rank test). Other comparisons were done using Mann–Whitney U-test and significant *P* values are shown: ***p < 0,001; **p < 0,01; *p < 0,05, as compared to healthy; *^#^p < 0,05*, as compared to No-ICU at the same time-point.

The bidimensional hierarchical gating strategy confirmed the results of the unbiased analysis (**Figure 3**). A significant increase in classical monocyte count was observed reaching significance in ICU patients at discharge (ICU D = 599, 456–740; HC = 349, 253–519 cells/µl, p = 0.04). A global decrease in non-classical monocyte population counts was observed in comparison with HCs (34, 24–43 µ/ml) in all patients at both time points (No-ICU HA/D = 6, 0.7–8.6 cells/µl, p = 0.007/13, 5–15 cells/µl, p = 0.05; ICU A/D = 2.7, 1–4.6 cells/µl, p = 0.0005/7, 3–8 cells/µl, p < 0.0001). No significant alterations were reported in the intermediate monocyte subset counts (**Figure 3A**).

Concerning the M2-like/immuno-regulatory marker expression, in the ICU group at discharge, a significant increase in CD163 MFI (HCs = 8,142, 7,280–9,945; ICU D = 14,938, 12,035–15,700, p = 0.01) and a tendency to increase in the percentages of CD14++CD16− monocytes showing M2-like markers (CD204+CD206+ classical monocytes at ICU D = 27, 16–33% *vs* HCs = 13, 9–17%, p = 0.08) was observed, complemented by the expansion of a HLA-DRhighPD-L1+ cell population (ICU D = 1.7, 0.7–2.8%; HCs = 0.6, 0.1–0.8%) (**Figure 3B**).

Concerning non-classical monocytes, the most striking feature in all patients from admission was the persistent decline in the frequency of the non-classical monocytes expressing Slan (HCs = 16, 7–25%, No-ICU HA/D = 1.6, 1.3–2.1%, p = 0.002/0.1, 0.1–0.6, p = 0.0009; ICU A/D = 0.4, 0–2, p = 0.0005/0.6, 0.2–0.7, p = 0.001), and the expansion of cells lacking the co-stimulatory molecules CD80 and CD86 at discharge (ICU = 26, 8–30%, HCs = 4, 0.9–9%, p = 0.01) (**Figure 3C**).

**Figure 4:**
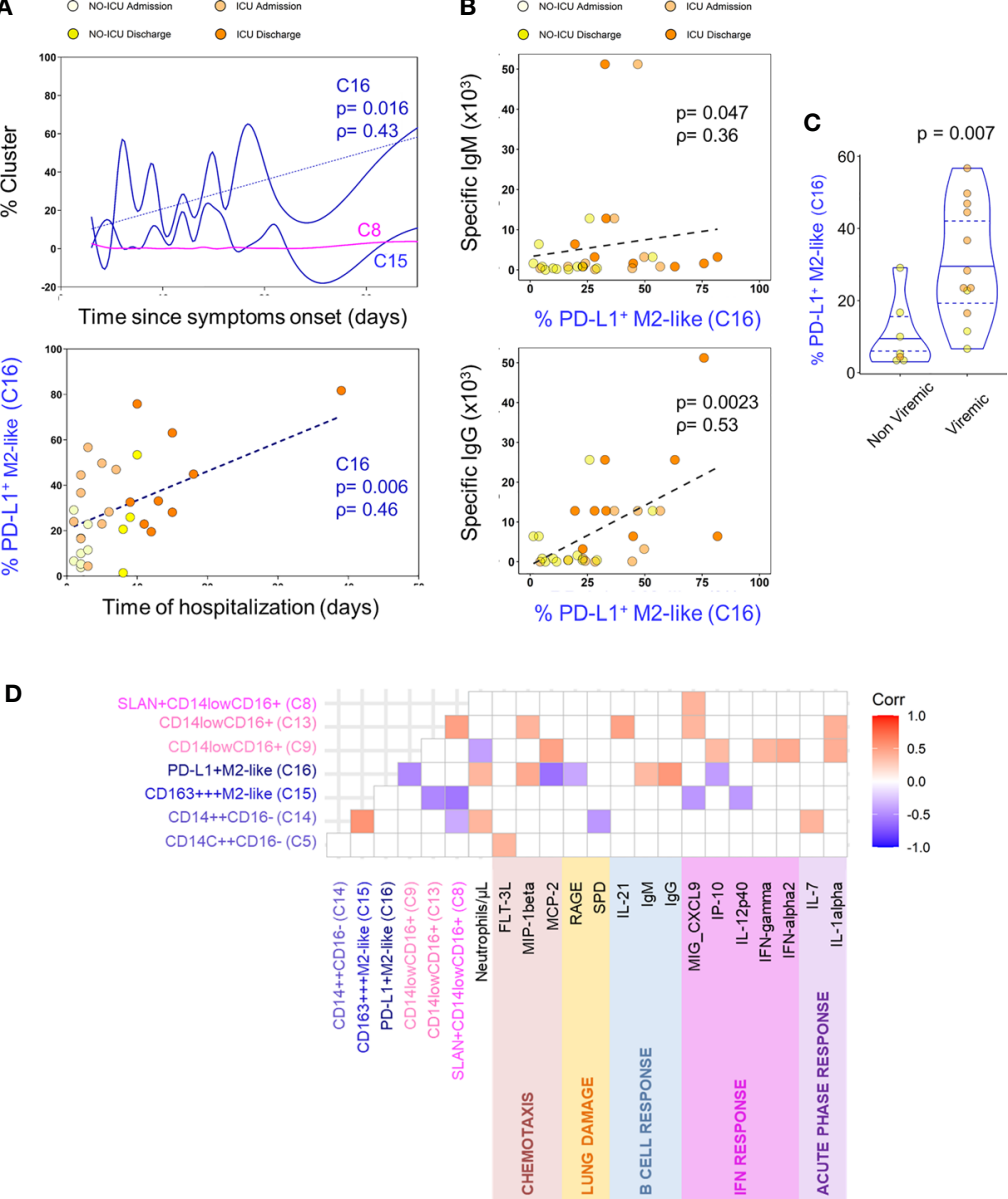
M2-like monocytes expanded until discharge and correlated with the decrease of inflammatory analytes. **(A)** Correlation of indicated cluster frequencies with days since symptoms onset (top) and time of hospitalization (bottom). **(B)** Correlation between PD-L1+M2-like cluster frequency and anti-SARS-CoV-2 specific IgM (top) and IgG (bottom) titers. **(C)** Frequency of PD-L1+M2-like cluster at admission in viremic *versus* non-viremic patients; comparison done using Mann-Whitney U-test and P value are shown. **(D)** Correlation matrix identifying the relation between monocyte X-shift clusters and serum markers with only significant correlations showed (p-value <0.05); Spearman Rank correlation coefficient were used.

Possible associations of relevant circulating myeloid cell populations with clinical variables and circulatory inflammatory or immune-regulatory mediators were evaluated. PD-L1+M2-like classical monocytes (C16) constituted the only population which frequency was positively correlated with the time elapsed from start of the symptoms and from hospital admission (**Figure 4A**). PD-L1+M2-like classical monocyte cluster percentage was also directly correlated with the anti-SARS-CoV-2 IgM and IgG levels (**Figure 4B**). This finding suggests that specific humoral immunity is developed in parallel with the significant increase of myeloid cell subsets with immunoregulatory phenotype, raising the possibility that the emergence of M2-like phenotype and the development of specific antibodies might be sustained by common mechanisms. Given the previous data on the impact of SARS-CoV-2 on myeloid cell differentiation (10–12), we also sought to evaluate if viremia might be a factor underlying the expansion of PD-L1+ M2-like classical monocytes (C16) and observed that their levels at admission were higher in patients with detectable SARS-CoV-2 viral load (**Figure 4C**). No other cell population was affected by the viremic status of the patients.

Moreover, as displayed in **Figure 4D**, the expansion of PD-L1+M2-like (C16) and CD163+++M2-like (C15) classical monocytes showed an inverse correlation with the levels of circulating inflammatory cytokines and chemokines related to the IFN pathway (IP-10, CXCL9, IL-12p40). Conversely, the non-classical monocyte populations (C8, C9, and C13) declined in parallel with several inflammatory cytokines, being correlated with the levels of MIG-CXCL-9, MCP-2, IFNα2, IFN*γ*, IL-1α, IP-10, IL-21, and MIP1-β (**Figure 4D**).

**Figure 5:**
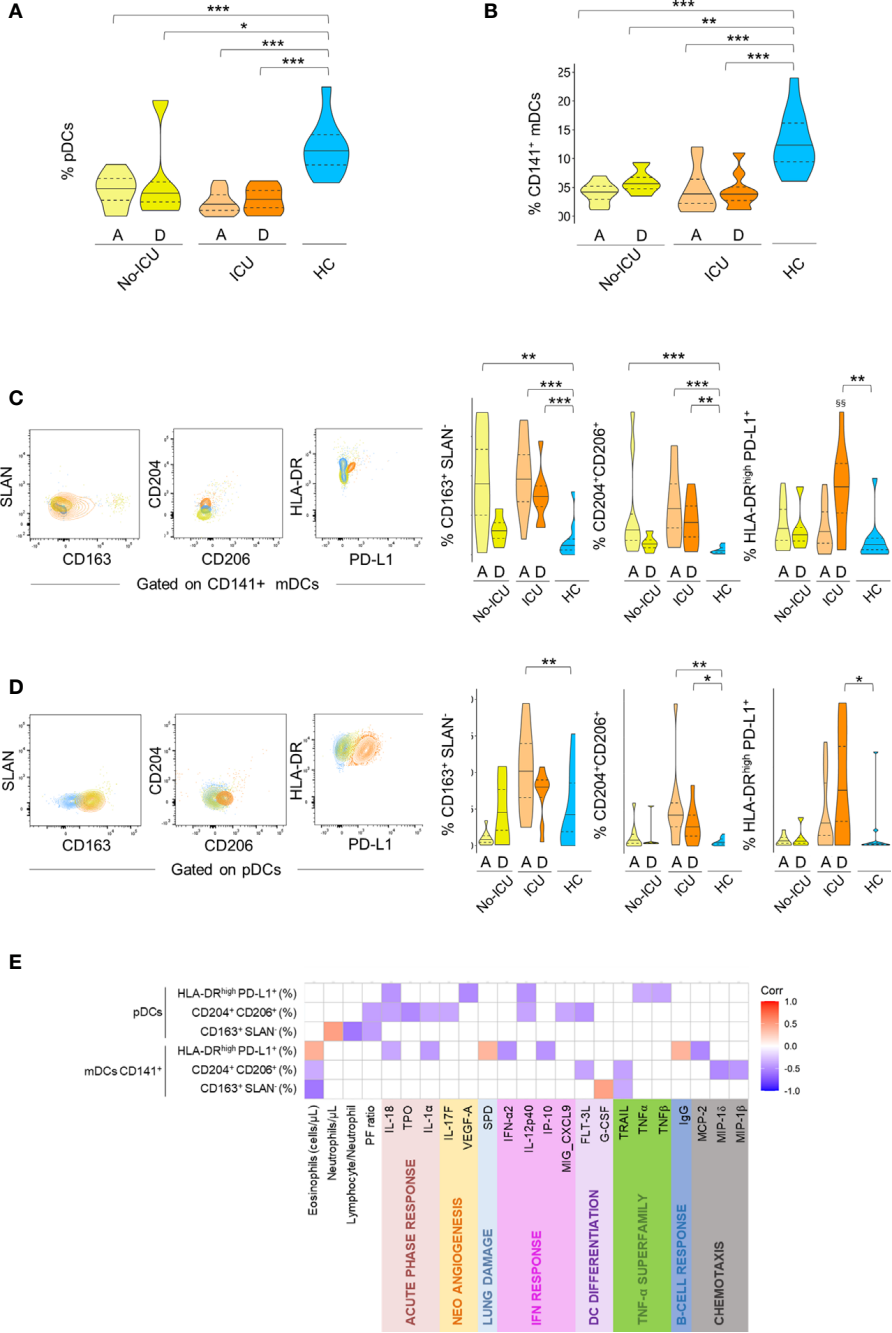
Immune regulatory phenotype of dendritic cells in severe COVID-19. Frequency of pDC cluster **(A)** and CD141+mDC cluster **(B)** identified by X-Shift within total CD45+Lin− cells in the different groups. (**C–D)** Illustrative dot-plots (left) of bi-dimensional hierarchical gating strategy were used to further analyze the phenotype of CD141+mDCs **(C)** and pDCs **(D)** from No-ICU (yellow) and ICU (orange) patients at discharge and healthy control (blue) and the respective graphs (right). Wilcoxon matched-pairs signed rank test to the paired analysis of the two-time points and significant P values are shown: *^§§^p < 0,01.* Mann–Whitney U-test were used for comparison with healthy controls: ***p < 0,001; **p < 0,01; *p < 0,05. **(E)** Correlation matrix identifying relations between frequency of the identified populations within pDCs and CD141+mDCs and serum markers from both time-points and from patient and control groups; Spearman Rank correlation coefficient was used and p < 0.05 are showed.

Concerning DC subsets, a persistent and marked contraction of pDCs (C1) and CD141+mDCs (C7) was observed in all patients, with a partial recovery at discharge in the No-ICU group (**Figures 5A, B**, respectively, and **Supplementary Table 2**).

A targeted analysis performed by manual gating, confirmed the contraction of all main DC populations (**Supplementary Figure 3**), and revealed a profile remarkably similar to the one observed in the monocyte compartment. Namely, an increase in the expression of scavenger receptors was observed in both CD141+ mDCs and pDCs from all patients, persisting until discharge in ICU patients (**Figures 5C, D**). ICU patients also featured an increased percentage of HLA-DRhighPD-L1+ cells in both DC populations at discharge, and a significant increase of CD163+ pDCs at ICU admission (**Figures 5C, D**).

If analyzing both patient groups and time points, the frequencies of HLA-DRhighPD-L1+ and CD204+CD206+ cells in pDCs and CD141+mDCs were inversely correlated with analytes related to IFN pathway, as well as to acute phase response proteins and TNF levels (**Figure 5E**), overall confirming the induction of an immune-modulatory signature also in DC sub-populations. No significant changes were reported for CD1c+ mDCs (**Supplementary Figure 4**).

**Figure 6:**
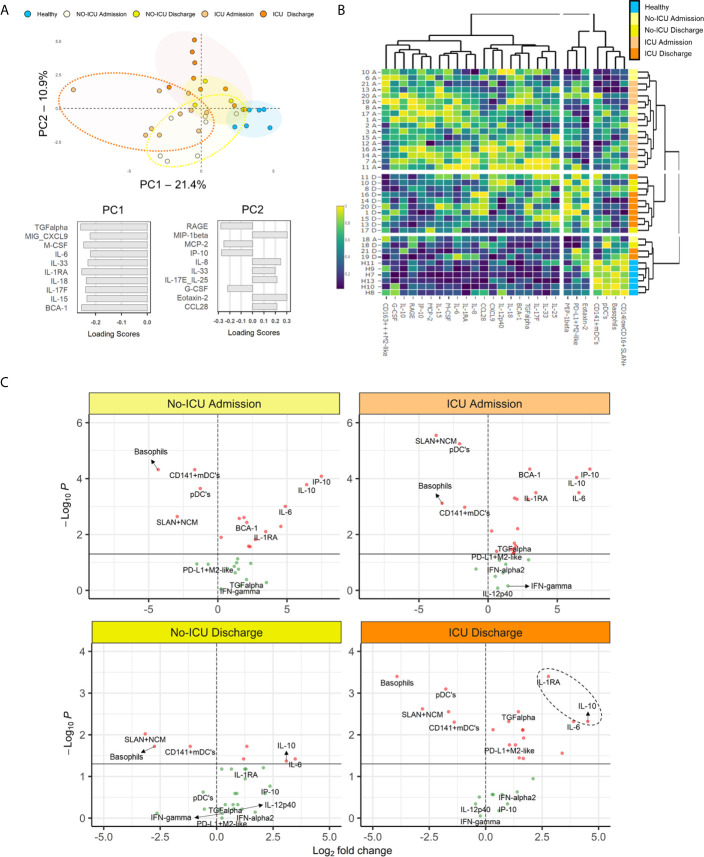
Myeloid cell populations and inflammatory/immunoregulatory serum markers segregate COVID-19 stages. **(A)** Principal component analysis (PCA) of the 35 serum analytes showed to have significant different levels as compared to healthy or between the time-points analyzed; loading scores of principal component (PC)1 and PC2 showing the top 10 highest absolute values. **(B)** Heatmap performed using the top 10 parameters in the PC1 and PC2 of the PCA analysis showed in **(A)** and the frequencies of the X-shift clusters found to be significantly altered in COVID-19 patients; dendrograms illustrate the hierarchical clustering; a color code was added to identify individual groups. **(C)** Volcano plots comparing the variables used in the heatmap showed in **(B)** in patient groups and healthy controls; p < 0.05 were considered significant.

For assessing the circulating cytokine environment, from the 71 inflammatory mediators analyzed, we selected 42 analytes showing significant changes between patients and HCs or within patient groups, or previously reported in literature as central for the hyperinflammatory syndrome pathogenesis (**Supplementary Table 3**) (5). Then, we evaluated using principal component analysis, the most relevant analytes for the segregation of patients from HCs (**Figure 6A**).

Subsequently, combining the analytes with higher loading scores in the principal components with the relevant myeloid cell populations described above, we were able to discriminate the admission and discharge datasets, as well as HCs, using unsupervised hierarchical clustering (**Figure 6B**).

Finally, using volcano plots, we showed that only ICU patients kept at discharge statistically significant increased levels of IL-1RA, IL-10, and IL-6 in comparison with HCs, in parallel with the expansion of myeloid cells expressing M2 markers and PDL-1 (**Figure 6C** and **Supplementary Table 3**).

Notably, in the present cohort, no significant increase was reported for IFN-γ, IL-1α, and IL-1β levels in all patients, in comparison to HCs. Moreover, although not significantly increased at admission, IFN-γ and IL-12p40 showed a negative correlation with time, declining from admission to discharge, in ICU patients (**Figure 6C** and **Supplementary Table 2**).

Altogether, our results show an immune-regulatory profile shift in myeloid cell populations in a cohort of severe patients surviving SARS-CoV-2. Moreover, the evolution towards recovery, in ICU patients, was linked to the expansion of a PD-L1+M2-like classical monocyte and DC subset, in parallel with control of SARS-CoV-2 plasma viral load, development of high titers of specific Ig, and a cytokine signature defined by persistently low IFN-γ, IL1-α, and IL1-β and high IL-1RA and IL10 levels (**Figure 6C**).

## Discussion

This longitudinal study revealed systemic immune-regulatory myeloid cell responses in all COVID-19 patients with respiratory insufficiency throughout the path to recovery.

Interestingly, our longitudinal data showed that the myeloid cell subpopulation modifications were associated with significant changes in the balance of pro-inflammatory and immune-regulatory cytokine/chemokine levels, in which low levels of IFN-α2, TNF-α, IL-1α, and IL-1β, decline of IFN-*γ* and IL-12p40, and persistence of significantly high IL-10, and IL-1RA were main features. Also, the evaluation of patients at admission and recovery allowed us to describe, in those admitted to ICU, the expression of immuno-regulatory elements, especially at discharge, whereas this profile was mainly present at admission to hospital in patients that did not require ICU. These findings support a contribution of the timely acquisition of a myeloid cell immune-regulatory phenotype to the recovery from respiratory insufficiency.

As already described in severe COVID-19, global reduction of pro-inflammatory myeloid cell subsets was observed in all patients in comparison to healthy subjects (10). It appears plausible that blunted type I interferon and IL-1 responses, together with high systemic levels of regulatory cytokines, support the M2-like differentiation observed in our COVID-19 patient cohort.

Circulating monocytes differentiate along a continuous gradient of phenotype states to macrophage-like cells and they are also precursors of myeloid DCs in tissues (19).

Notably, healthy resident alveolar macrophages show an immune-regulatory/M2-like phenotype, favoring the continuous non-inflammatory clearance of pathogens, debris, and apoptotic cells. However they also secrete the cytokines and chemokines that orchestrate the recruitment of inflammatory bone marrow derived cells in the course of infections (20). Regarding myeloid cell phenotype role in viral infection prognosis, a M1-like shift, correlated with secretion of cytokines like IFN-γ, TNF-α, IL-6, and IL-12, both in mucosal associated and in systemic myeloid cells, was considered determinant for worse outcomes in life-threatening viral infection (21, 22). However, viruses, *per se*, can also divert macrophage phenotype towards M2-like, for instance through increasing the production of cytokines like IL-4 or IL-10 (21).

In the context of an acute viral infection, it is particularly remarkable the extreme decline in the Slan+ subset. A very recent paper, performed on hospitalized COVID-19 patients, showed significantly increased sCD163 and sCD14 and a reduction of non-classical monocytes, mDCs and pDCs in COVID-19. However, in this study, the Slan positive population was classified within DCs, while our unbiased flow cytometry data analysis, as well as previous evaluations, performed through genome-wide transcriptional profiling, demonstrated that those cells cluster together with non-classical monocyte population (22–25).

The non-classical monocyte subset was shown to expand in several bacterial and viral infections (26–28). Nevertheless, there are reports of a reduction of this sub-population in viral infections and in inflammatory or auto-immune diseases, where the decrease in circulation was mainly attributed to tissue migration (29–31). Interestingly, inflammasome activation and pyroptotic cell death was described in circulating monocytes from severe COVID-19 patients and non-classical monocytes might be among the first cells to undergo this lytic programmed cell death process, after inflammasome engagement (32).

Importantly, in our prospective cohort of patients surviving severe COVID-19, the significant reduction of Slan+ non-classical monocytes was associated to a contemporaneous general myeloid cell shift towards a M2-like phenotype. Indeed, a similar transition from a pro- to an anti-inflammatory status of human monocytes was described to enhance protective functions like phagocytosis, anti-microbial activity, and tissue remodeling, during sepsis (33).

In a recent *in-vitro* study, it was demonstrated that SARS-CoV-2 infection of monocytes and macrophages is abortive and associated with secretion of IL-10 and TGF-β immunoregulatory cytokines, inducing a transcriptional program characterized by the upregulation of M2 molecules (34).

In addition, a longitudinal study on cytokine and chemokine response signature in severe COVID-19 demonstrated an increase in multiple type 2 effectors, including IL-5, IL-13, IgE, with low expression of pro-inflammatory cytokines and enrichment in tissue repair genes in recovering patients, while higher interferons and pro-inflammatory cytokines and chemokines were observed in patients with worse prognosis (35).

It is relevant that we were able to quantify SARS-CoV-2 plasma viral load in a significant number of patients at admission, particularly in those that required ICU. Moreover, the presence of viral genes was associated with increased IL-10 levels and expansion of the PD-L1+M2-like monocytes, suggesting a role for SARS-CoV-2 viral load in induction of immune-regulatory changes in the innate immune system.

Besides M2-like polarization in myeloid cells, previous data on classical monocytes expressing several scavenger receptors and PD-L1 (PD-L1+M2-like) in viral infections are scarce. Similar subsets have been studied mainly in association with the immune-suppressive effects of neoplastic processes. Interestingly, they are considered to be induced by commensal bacteria with beneficial immunomodulatory properties in inflammatory diseases (36). Therefore, a favorable role for this particular cell subset in immune-regulation during viral infections should be further assessed.

Alterations attributable to immuno-regulatory/suppressive changes of myeloid cell compartment, such as reduction of HLA-DR or increase of CD163 in classical monocytes, and low levels of non-classical monocytes, were defined as characteristics of the pathogenic mechanism of immune-paralysis (37–40). On the other hand, a hyper-inflammatory syndrome associated with cytokine storm was shown to be linked with critical disease and fatality in severe viral infections such as Ebola, Dengue, or Influenza A H1N1 (41–43). Furthermore, in severe COVID-19, the increase in pro-inflammatory cytokines and chemoattractant proteins was associated with fatal outcome (44–50).

SARS-CoV-2 was demonstrated to influence innate immune responses in a complex way, inducing exuberant inflammatory cytokine production associated with weak type I and III IFN responses and reduced IFN stimulated gene (ISG) expression, as previously observed for SARS-CoV-1 and MERS-CoV (51). The function of all the 30 proteins encoded by the SARS-CoV-2 genome was recently studied, demonstrating that several non-structural proteins have a direct suppressive effect on IFN signaling (52).

A highly impaired IFN-α and β response was associated with persistent SARS-CoV-2 viremia and exacerbated inflammation in severe and critically ill patients (53). The importance of a reduced IFN response in SARS-CoV-2 infection was highlighted also by two studies showing that inborn errors of immunity involving IFN type I signaling pathways or auto-antibodies against type I IFNs have increased frequency in patients developing severe and critical disease (54, 55).

Conversely, strong/rapid type I IFN responses and distinctive myeloid cell signatures were described in early, mild, and moderate SARS-CoV-2 infection (11, 12, 56).

Our data supports the idea that IFN response or prophylactic treatment with recombinant IFN can have different roles depending on the timing: an early and robust increase might be needed for a rapid viral clearance in the initial stage of SARS-CoV-2 infection (57, 58). The present patient cohort might be considered as representative of a late phase, characterized by persistent viral replication, inducing systemic spreading of the virus and high levels of numerous inflammatory mediators. In this disease stage, a coordinated action of low IFNs levels, high immune-regulatory cytokines/decoy receptors and immuno-suppressive/regulatory cell differentiation in circulation, might induce the interruption of the auto-amplifying inflammatory process. Immune-regulatory responses may re-establish an equilibrium in the host-pathogen interaction, favoring recovery from the respiratory insufficiency.

In this context, M2-like myeloid cells could represent potential cellular targets contributing the required negative feedback to the inflammatory response.

At the same time, our data raise concerns regarding evolution towards lung fibrosis in COVID-19, since the expression of M2 phenotype markers in macrophages was associated with pathogenesis of the fibro-proliferative process in lung fibrosis, both idiopathic and associated to auto-immune conditions (59, 60). Although early studies in severe COVID-19 patients linked extended lung fibrosis to the high pressure ventilation-related barotrauma, the last reports under protective ventilation still show presence of fibrotic abnormalities, reduced health-related quality of life, and persistent respiratory symptoms (61). Consequently, long-term studies are necessary for the evaluation of myeloid cell phenotype in circulation, bronco-alveolar lavage, and lung tissue, to address the possible persistence of M2 signature and its relationship with long-term sequelae in severe COVID-19.

Some limitation of the study should be noted, mainly related to the relatively small sample size. In agreement with the gender bias in severe COVID-19, few female patients were admitted to hospital at the time of the study, precluding the evaluation of the effect of sex as a variable.

Additionally, it was not possible to evaluate the effect of the treatment, however it has to be considered that numerous severe COVID-19 patients were enrolled before the routine administration of steroids was implemented. In fact, a particular importance has to be given to the possible impact of a treatment with steroids on M2-like phenotype induction. Steroids were demonstrated to influence myeloid cell phenotype both *in-vivo* and *in-vitro* (62, 63). However, the results of those studies are not conclusive, and it was not the purpose of the present research to exclude an impact of immune-regulatory drugs on cell phenotype in patients surviving severe COVID-19. Steroids have been demonstrated to have a positive impact on severe disease outcome, and the induction of a M2-like phenotype in myeloid cells could possibly concur to this therapeutic effect (64). Nevertheless, the development of larger perspective studies, exploring the possible impact of a treatment with steroids on regulatory myeloid cell phenotype induction in recovery from symptomatic COVID-19, would be of critical significance.

Finally, even if high mortality is registered in critically ill COVID-19 patients, only two deaths were observed in the patient cohort, not providing statistical power for the evaluation of a possible effect of innate immune system signature on death rate.

In conclusion, the results of the present study support that severe COVID-19 recovery is associated with timely acquisition of a myeloid cell immune-regulatory phenotype. Consequently, the development and use of therapeutic agents, in addition to glucocorticoids, that would favor the immune-regulatory shift of innate immune system components would be the best strategy at this disease stage.

## Data Availability Statement

The raw data supporting the conclusions of this article will be made available by the authors, without undue reservation.

## Ethics Statement

The studies involving human participants were reviewed and approved by the ethics committee at the CHULN/Faculdade de Medicina da Universidade de Lisboa. The patients/participants provided their written informed consent to participate in this study.

## Author Contributions

ACT, SMF, and AES designed the study. CM and SF enrolled and followed up the patients. ACT, GBF, AMCG, AG-S, PR, CMC, JL, DFS, and ARMA performed the experimental research. AG and MS performed the antibody assays. MV supervised specific antibody titers quantification. ACT and GBF performed the supervised and unsupervised analysis and the statistical analysis. ACT wrote the manuscript. All authors participated to data discussion and manuscript revision. All authors contributed to the article and approved the submitted version.

## Funding

The Research was funded by Fundação para a Ciência e Tecnologia (FCT), “APOIO ESPECIAL RESEARCH 4COVID-19” projects 803, 125, 231_596873172, and 729. AMCG and GF received fellowships funded by FCT (DOCTORATES4COVID-19, 2020.10202.BD), and JANSSEN- CILAG FARMACÊUTICA, respectively. The funder was not involved in the study design, collection, analysis, interpretation of data, writing of the article or decision to submit it for publication. MV was supported by the European Union H2020 ERA project (No 667824 – EXCELLtoINNOV). This project has received funding from the European Union’s Horizon 2020 research and innovation programme under grant agreement No 667824.

## Conflict of Interest

The authors declare that the research was conducted in the absence of any commercial or financial relationships that could be construed as a potential conflict of interest.
