# Supplementary figures and images for: Severe COVID-19 Recovery Is Associated with Timely Acquisition of a Myeloid Cell Immune-Regulatory Phenotype
Source: Front Immunol. 2021 Jun 23;12:691725. doi: 10.3389/fimmu.2021.691725 (PMC8265310; doi:10.3389/fimmu.2021.691725)

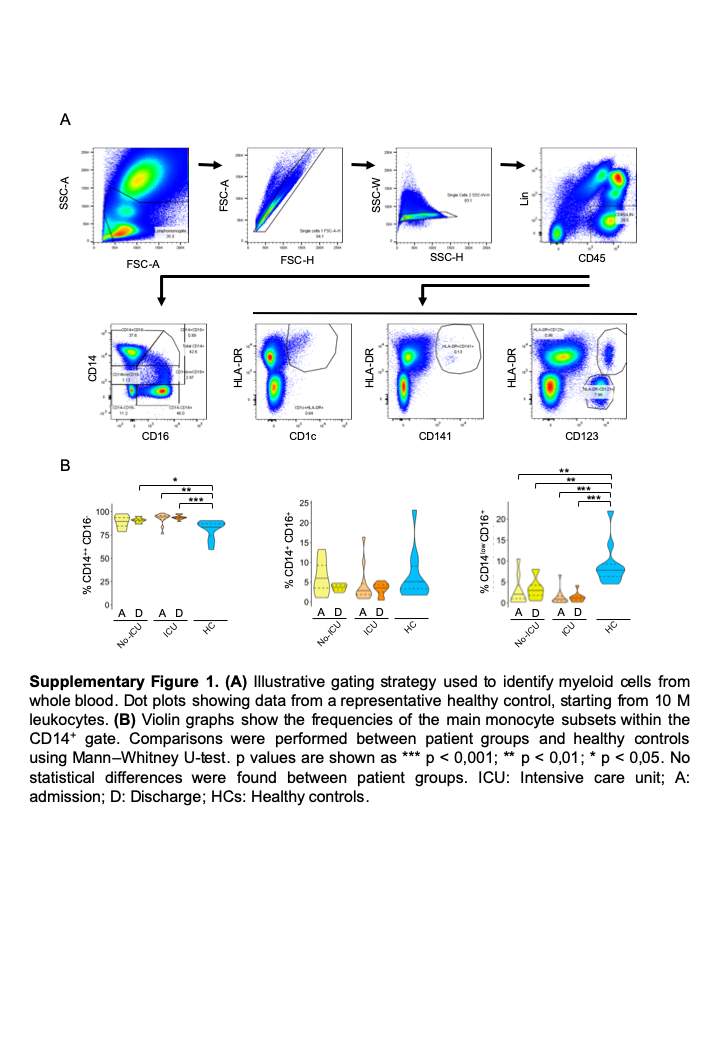

Supplement: Supplementary file 1 [file Image_1.tiff]

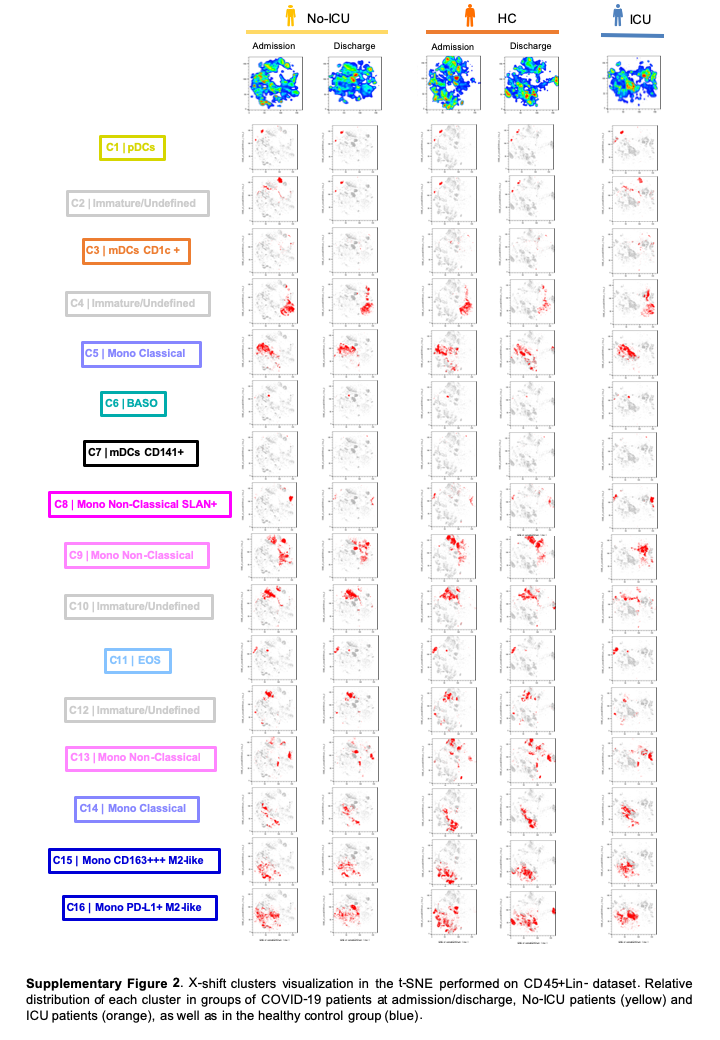

Supplement: Supplementary file 2 [file Image_2.tiff]

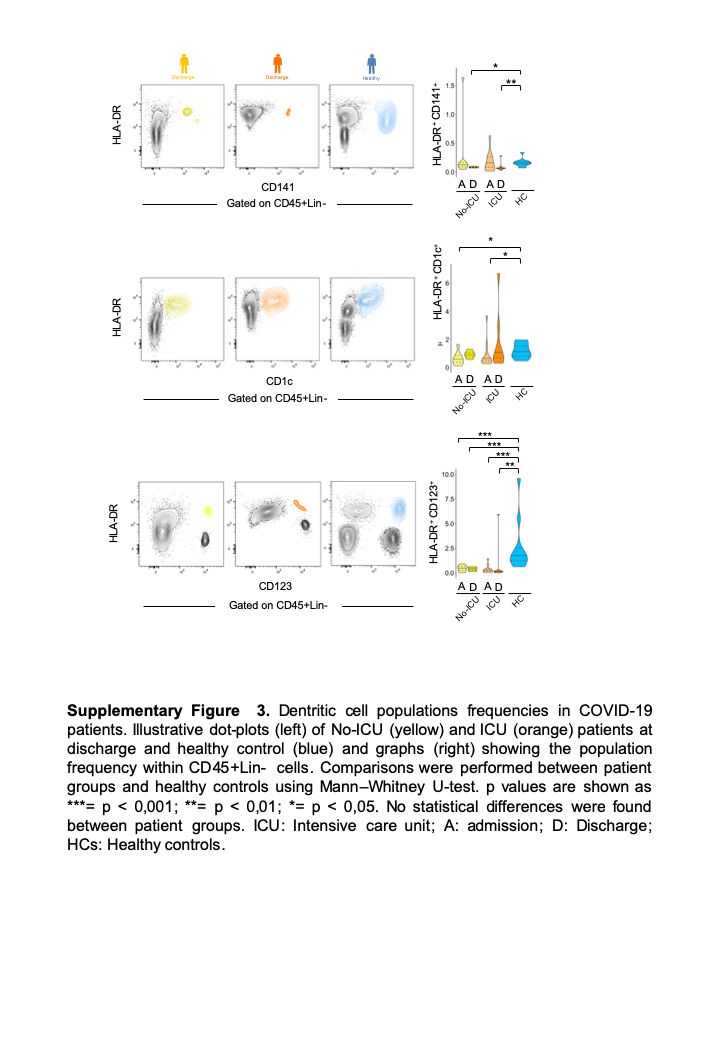

Supplement: Supplementary file 3 [file Image_3.tiff]

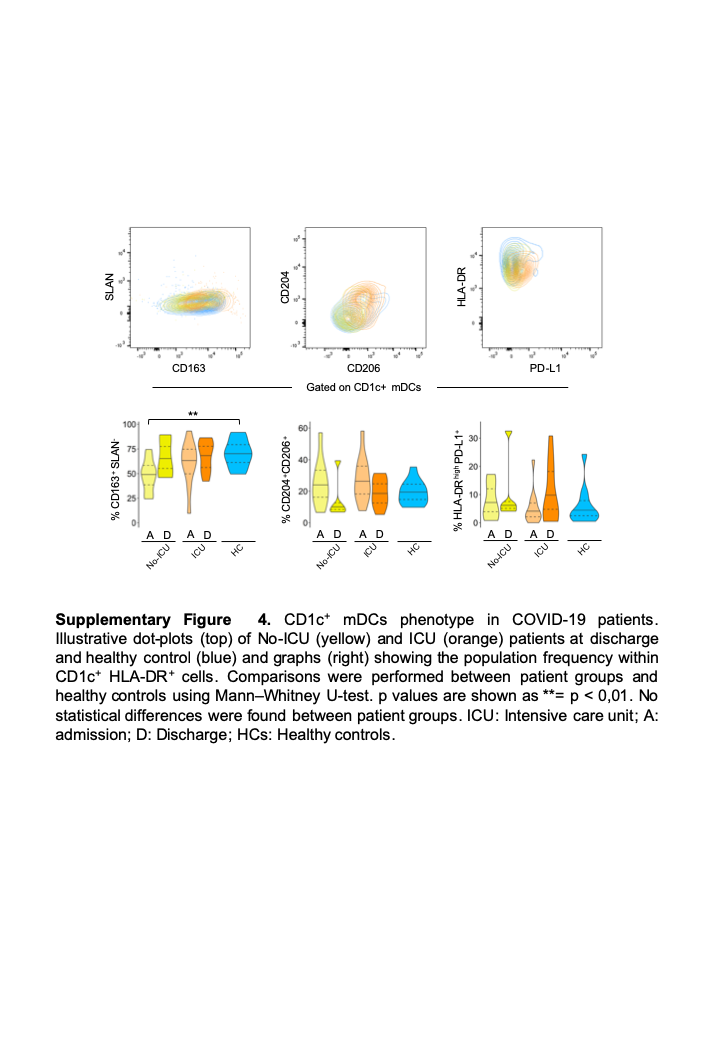

Supplement: Supplementary file 4 [file Image_4.tiff]
