# Supplementary material for: Severe COVID-19 Recovery Is Associated with Timely Acquisition of a Myeloid Cell Immune-Regulatory Phenotype
Source: Front Immunol. 2021 Jun 23;12:691725. doi: 10.3389/fimmu.2021.691725 (PMC8265310; doi:10.3389/fimmu.2021.691725)
Supplement: Supplementary file 5 [file Table_1.docx]

**Supplementary Table 1. Flow-cytometry panel.**

|  | Marker | Company | Clone |
| --- | --- | --- | --- |
| Vio Blue | Slan | Milteny Biotec | DD-1 |
| BV510 | CD141 | BD Biosciences/BD Horizon | 1A4 |
| BV605 | CD45 | BD Biosciences | HI30 |
| BV650 | HLA-DR | Biolegend | L243 |
| BV711 | CD86 | Biolegend | IT2.2 |
| BV785 | PD-L1 | Biolegend | 29E2A3 |
| Fitc | CD3 | eBioscience/Invitrogen | αT3 |
| Fitc | CD19 | eBioscience/Invitrogen | HB19 |
| Fitc | CD66b | Biolegend | G10FS |
| Percp Cy5.5 | CD14 | eBioscience/Invitrogen | 61D3 |
| Pe | CD80 | Biolegend | 2D10 |
| Pe Dazzle-594 | CD163 | Biolegend | GH1/61 |
| Pe-Cy5 | CD206 | Biolegend | 15-2 |
| Pe-Cy7 | CD123 | eBioscience | 6H6 |
| Apc | CD204 | Biolegend | 7C9C20 |
| Af700 | CD16 | Biolegend | 368 |
| Apc-Cy7 | CD1c | Biolegend | L161 |
