# Supplementary material for: Severe COVID-19 Recovery Is Associated with Timely Acquisition of a Myeloid Cell Immune-Regulatory Phenotype
Source: Front Immunol. 2021 Jun 23;12:691725. doi: 10.3389/fimmu.2021.691725 (PMC8265310; doi:10.3389/fimmu.2021.691725)
Supplement: Supplementary file 6 [file Table_2.docx]

**Supplementary Table 2. X-Shift cluster frequency in the CD45+Lin- dataset.**

| CLUSTERS | ANNOTATION | No-ICU | ICU | HCs | p  (ICU  vs HCs )^a^ | p  (No-ICU vs HCs)^a^ | p  (ICU vs  No-ICU)^a^ |
| --- | --- | --- | --- | --- | --- | --- | --- |
| 1. A   D | pDCs | 0.57 (0.19-0.84)  0.49 (0.14-1.49) | 0.22 (0.07-0.56)  0.33 (0.09-0.61) | 1.4  (0.9-1.6) | **<0.0001**  **<0.0001** | **0.0002**  **0.04** | 0.28  0.69 |
| 1. A   D | Immature/  Unclassified | 1.9 (1.7-6.3)  0.98 (0.54-2.3) | 1.8 (1-5.6)  1.1 (0.5-2.1) | 0.2  (0.04-0.5) | **0.0003**  **0.01** | **0.0004**  **0.03** | 0.28 |
| 1. A   D | mDCs CD1c+ | 0.13 (0.07-0.4)  0.05 (0.01-0.13) | 0.05 (0.02-0.12)  0.04 (0.02-0.13) | 0.13  (0.05-0.36) | **0.05**  **0.05** | 0.98  0.12 | 0.72  0.82 |
| 1. A   D | Immature/  Unclassified | 16 (6.5-34.7)  2 (0.1-19) | 0.03 (0.008-0.33)  0.07 (0.01-0.65) | 16.6  (2.4-45-8) | **<0.0001**  **<0.0001** | 0.86  0.14 | 0.7  0.18 |
| 1. A   D | Mono Classical | 11.3 (3.7-25.6)  11.1 (7.2-27.5) | 8 (3.4-10)  9 (7-14) | 8.5  (6.5-18.2) | 0.5  0.88 | 0.82  0.50 | >0.99  0.43 |
| 1. A   D | Basophils | 0.4 (0.01-0.93)  0.06 (0.2-0.8) | 0.002 (0-0.02)  0.003 (0-0.02) | 0.3  (0.001-1.39) | 0.07  **0.03** | 0.88  0.25 | 0.75  0.92 |
| 1. A   D | mDCs CD141+ | 0.04 (0.02-0.05)  0.05 (0.04-0.07) | 0.02 (0.01-0.06)  0.03 (0.02-0.05) | 0.11  (0.09-0.17) | **0.001**  **0.0005** | **<0.0001**  **0.005** | 0.28  0.18 |
| 1. A   D | Mono Non-Classical SLAN+ | 0.08 (0.04-1.6)  0.06 (0.02-0.8) | 0.09 (0.04-0.2)  0.21 (0.02-0.49) | 4.5  (3.1-6.3) | **<0.0001**  **<0.0001** | **0.002**  **0.005** | 0.48  0.87 |
| 1. A   D | Mono Non-Classical | 18.8 (9.4-32.6)  16.8 (11.2-38.6) | 10.7 (4.2-19)  12.3 (4.4-18.6) | 18.9  (11.6-24.2) | 0.17  0.13 | >0.99  >0.99 | >0.99  0.24 |
| 1. A   D | Immature/  Undefined | 2.5 (1.8-3.3)  3.7 (2.6-25.6) | 8.2 (5.2-13.7)  4.9 (3.2-11.5) | 2.5  (1.8-3.3) | **0.001**  **0.02** | 0.71  0.11 | >0.99  0.89 |
| 1. A   D | Eosinophils | 0.18 (0.07-0.54)  0.26 (0.05-1.76) | 0.22 (0.002-1.79)  0.07 (0.02-0.59) | 3.3  (1.7-5.7) | **0.0008**  **<0.0001** | **<0.0001**  **0.013** | **0.009**  0.36 |
| 1. A   D | Immature/  Undefined | 0.34 (0.08-1.17)  0.5 (0.3-9.6 | 0.33 (0.06-1.48)  0.5 (0.14-1.6) | 0.76  (0.51-0.98) | 0.33  0.60 | 0.34  0.44 | 0.6  0.82 |
| 1. A   D | Mono Non-Classical | 2.4 (0.63-3.6)  2.1 (0.9-8.6) | 3.3 (0.6-14)  4.2 (1.8-12.4) | 3.38  (2.5-6.6) | 0.89  0.55 | 0.13  0.66 | 0.6  0.45 |
| 1. A   D | Mono Classical | 0.87 (0.08-1.85)  0.15 (0.03-6.3) | 1.7 (0.09-6.7)  1.12 (0.001-15) | 0.84  (0.05-1.85) | 0.26  0.86 | 0.98  0.64 | >0.99  >0.99 |
| 1. A   D | CD163+++ M2like | 6.11 (0.8-14)  1.19 (0.11-4.25) | 5.9 (0.9-21)  3.5 (0.3-8.2) | 1.19  (0.11-4.25) | **0.04**  **0.04** | 0.10  0.58 | 0.35  0.41 |
| 1. A   D | PD-L1+ M2like | 9.9 (4.5-19)  20.6 (10.39) | 34 (23-56)  33 (25-69) | 22  (11.5-27.8) | **0.04**  **0.007** | 0.11  0.9 | 0.48  0.08 |
| Values expressed as medians (interquartile range). Comparisons made using Mann-Whitney U test. Significant differences in bold. ICU= Intensive care unit; HCs= Healthy Controls; A= Admission; D= Discharge.  ^a^ Comparisons performed using Mann-Whitney U test. | | | | | | | |
