# Supplementary material for: Severe COVID-19 Recovery Is Associated with Timely Acquisition of a Myeloid Cell Immune-Regulatory Phenotype
Source: Front Immunol. 2021 Jun 23;12:691725. doi: 10.3389/fimmu.2021.691725 (PMC8265310; doi:10.3389/fimmu.2021.691725)
Supplement: Supplementary file 7 [file Table_3.docx]

**Supplementary Table 3. Circulating cytokines, chemokines and decoy receptors.**

| Cytokine or  Chemokine | HCs | NO-ICU  A/D | | HCs vs NO-ICU p^a^  A/D | | ICU  A/D | | HCs vs ICU p^a^  A/D | | Days from symptoms^b^  ρ  p | Days in hospitalization^b^  ρ  p |
| --- | --- | --- | --- | --- | --- | --- | --- | --- | --- | --- | --- |
| CCL21  (6CKine) | 78  (64-879) | 131  (114-236) | 293  (216-345) | 0,13 | 0,27 | 396  280-434 | 330  137-399 | 0,003 | 0,046 | 0.021  0.911 | 0.021  0.910 |
| CXCL13  (BCA-1) | 19  (7-26) | 74  (62-85) | 22  (18-38) | <0,0001 | 0,48 | 118  (89-188) | 40  (28-63) | <0,0001 | 0,018 | -0.339  0.062 | -0.546  0.001 |
| CCL28 | 420  (389-435) | 498  (490-526) | 505  (485-520) | 0,01 | 0,07 | 520  (488-527) | 512  (488-555) | 0,007 | 0,008 | -0.012  0.948 | 0.037  0.844 |
| Eotaxin-2 | 745  (346-959) | 239  (97-418) | 752  (651-1532) | 0,06 | 0,61 | 348  (190-648) | 958  (775-1376) | 0,220 | 0,272 | 0.639  <0.001 | 0.543  0.002 |
| G-CSF | 5  (1-31) | 36  (25-48) | 1  (1-3) | 0,08 | 0,76 | 27  (21-32) | 10  (9-11) | 0,147 | 0,313 | -0.453  0.010 | -0.601  <0.001 |
| CXCL1  (GROα) | 19  (12-22) | 43  (38-84) | 35  (27-36) | 0,01 | 0,17 | 44  (33-57) | 41  (34-51) | 0,007 | 0,008 | -0.124  0.506 | -0.281  0.125 |
| IFN-α2 | 10  (9-24) | 50  (15-101) | 24  (9-104) | 0,05 | 0,72 | 50  (13-68) | 23  (19-132) | 0,052 | 0,235 | -0.023  0.656 | -0.083  0.903 |
| IFN-γ | 1.8  (0.6-6.2) | 2.5  (1.7-13.2) | 2.5  (0.6-5.7) | 0,28 | 0,76 | 4.4  (1.6-8.1) | 1.4  (0.6-3.9) | 0,368 | 0,890 | -0.251  0.173 | -0.391  0.030 |
| IL-1α | 9  (6-14) | 18  (11-33) | 18  (8-26) | 0,23 | 0,76 | 22  (11-27) | 25  (6-38) | 0,118 | 0,259 | -0.052  0.782 | -0.059  0.754 |
| IL-1β | 14 | 19  (84-35) | 27  (14-39) | 0,69 | 0,48 | 40  (19-48) | 27  (5-46) | 0,147 | 0,456 | 0.056  0.764 | 0.089  0.633 |
| IL1RA | 2  (2-4) | 13  (11-59) | 7  (5-10) | <0,0001 | 0,07 | 23  (12-57) | 12  (10-25) | <0,0001 | <0,0001 | -0.157  0.399 | -0.076  0.685 |
| IL-6 | 0.5  (0.2-0.9) | 18.5  (13.0-30.4) | 3.5  (2.2-8.8) | <0,0001 | 0,04 | 18.8  (4.7-48.4) | 3.8  (1.3-11.8) | <0,0001 | 0,005 | -0.407  0.023 | -0.379  0.035 |
| IL-7 | 4  (3-6) | 12  (7-19) | 10  (7-17) | 0,08 | 0,11 | 15  (8-20) | 4  (4-9) | 0,007 | 0,711 | -0.265  0.150 | -0.288  0.116 |
| IL-8 | 10  (6-16) | 20  (19-25) | 13  (9-16) | 0,03 | 0,61 | 18  (18-21) | 22  (18-44) | 0,011 | 0,012 | 0.011  0.953 | 0.058  0.757 |
| IL-10 | 0.3  (0.1-0.5) | 21.0  (4.9-38.9) | 2.7  (0.5-4.8) | <0,0001 | 0,04 | 16.7  (8.5-28.0) | 4.5  (3.2-9.7) | <0,0001 | 0,005 | -0.468  0.008 | -0.620  <0.001 |
| IL-12p40 | 42  (27-52) | 83  (55-146) | 80  (39-150) | 0,06 | 0,61 | 59  (28-102) | 34  (18-41) | 0,492 | 0,456 | -0.426  0.017 | -0.284  0.121 |
| IL-15 | 7  (6-7) | 21  (19-29) | 19  (16-19) | <0,0001 | 0,02 | 28  (21-30) | 19  (18-21) | <0,0001 | 0,008 | -0.229  0.216 | -0.255  0.165 |
| IL_17E  (IL-25) | 105  (39-144) | 175  (124-251) | 165  (85-255) | 0,12 | 0,48 | 221  (211-473) | 219  (117-353) | 0,007 | 0,113 | 0.028  0.880 | 0.094  0.614 |
| IL-17F | 5  (5-6) | 8  (6-16) | 8  (6-11) | <0,0001 | 0,07 | 11  (7-18) | 13  (6-24) | <0,0001 | 0,008 | -0.042  0.821 | 0.005  0.978 |
| IL-18 | 15  (7-24) | 79  (47-129) | 48  (17-97) | <0,0001 | 0,26 | 103  (72-151) | 26  (25-59) | 0,001 | 0,036 | -0.116  0.533 | -0.281  0.125 |
| IL-21 | 2  (2-3) | 3  (2-3) | 3  (3-3) | 0.296 | 0.105 | 4  (3-6) | 3  (3-4) | 0.018 | 0.036 | 0.076  0.683 | 0.120  0.519 |
| IL-22BP | 22070  (19461-22626) | 27057  (18026-48263) | 38324  (16948-60111) | 0,28 | 0,76 | 28844  (25089-41253) | 43590  (36807-57165) | 0,073 | 0,001 | 0.156  0.401 | 0.127  0.497 |
| IL-27 | 1584  (744-2142) | 2509  (2057-3380) | 1682  (894-2577) | 0,06 | 0,76 | 4340  (2948-6839) | 2419  (2069-3148) | 0,001 | 0,224 | -0.138  0.460 | -0.111  0.554 |
| IL-33 | 2  (1-2) | 3  (2-57) | 9  (2-29) | 0,27 | 0,06 | 18  (3-50) | 27  (3-104) | 0,011 | 0,028 | 0.069  0.714 | 0.208  0.261 |
| IP-10 | 35  (28-47) | 1761  (963-8609) | 185  (98-275) | <0,0001 | 0,17 | 1469  (976-5571) | 48  (27-85) | <0,0001 | 0,456 | -0.631  <0.001 | -0.767  <0.001 |
| MCP-2 | 20  (20-22) | 102  (84-485) | 27  (23-55) | <0,0001 | 0,07 | 84  (44-90) | 23  (21-28) | 0,007 | 0,272 | -0.594  <0.001 | -0.672  <0.001 |
| M-CSF | 52  (27-71) | 264  (160-307) | 148  (102-173) | 0,02 | 0,11 | 222  (143-282) | 206  (113-222) | 0,016 | 0,037 | -0.236  0.201 | -0.230  0.214 |
| CCL-22  MDC | 899  (763-1239) | 692  (442-786) | 521  (457-585) | 0,11 | 0,01 | 342  (261-599) | 282  (224-411) | 0,002 | 0,005 | -0.391  0.030 | -0.311  0.088 |
| MIG  CXCL9 | 1044  (976-1261) | 1515  (1176-2530) | 2354  (1593-3392) | 0,14 | 0,04 | 2740  (1413-5231) | 1531  (1086-1814) | 0,031 | 0,272 | -0.211  0.255 | -0.172  0.355 |
| MIP-1β | 21  (17-27) | 24  (18-46) | 52  (42-58) | 0,75 | 0,26 | 34  (29-45) | 54  (44-60) | 0,073 | 0,005 | 0.359  0.047 | 0.428  0.016 |
| MIP-1δ | 821  (603-866) | 3578  (2926-4408) | 2476  (1351-3619) | 0,02 | 0,01 | 1275  (1021-2731) | 2676  (2382-3353) | 0,016 | <0,0001 | -0.023  0.902 | 0.130  0.487 |
| PDGF-AA | 2255  (1797-2346) | 3780  (3027-4088) | 3271  (2557-4463) | <0,0001 | 0,11 | 3779  (3311-4174) | 3642  (2669-5058) | <0,0001 | 0,036 | -0.128  0.494 | -0.143  0.441 |
| RAGE | 146  (114-184) | 411  (263-443) | 250  (144-365) | <0,0001 | 0,26 | 431  (373-668) | 47  (27-72) | <0,0001 | 0,003 | -0.684  <0.001 | -0.703  <0.001 |
| SDF-1α+β | 2497  (1674-3652) | 1919  (1845-2097) | 3110  (1732-4635) | 0,92 | 0,76 | 1576  (804-1805) | 1815  934-2697 | 0,042 | 0,549 | 0.079  0.671 | 0.065  0.727 |
| SPD | 768  (6-1732) | 1374  (861-2178) | 5432  (3416-7404) | 0,29 | 0,01 | 4281  (1227-8091) | 1632  (603-6699) | 0,022 | 0,143 | -0.033  0.859 | -0.027  0.884 |
| TARC | 25  (19-28) | 17  (10-21) | 23  (15-28) | 0,14 | 0,91 | 14  (7-17) | 28  (12-30) | 0,022 | 0,864 | 0.166  0.371 | 0.243  0.188 |
| TGF-α | 9  (8-10) | 17  (15-28) | 12  (9-15) | 0,05 | 0,48 | 34  (18-37) | 21  (12-31) | 0,002 | 0,003 | 0.033  0.859 | -0.007  0.971 |
| TNF-α | 44  (26-151) | 125  (100-176) | 113  (40-184) | 0,18 | 0,76 | 171  (109-186) | 61  (45-162) | 0,171 | 0,607 | -0.256  0.165 | -0.424  0.018 |
| TNF-β | 12  (9-20) | 14  (10-44) | 31  (22-40) | 0,41 | 0,26 | 21  (19-40) | 17  (13-19) | 0,263 | 0,689 | -0.163  0.380 | -0.221  0.232 |
| TPO | 249  (242-279) | 762  (561-1247) | 547  (230-910) | 0,02 | 1 | 744  (413-1206) | 958  (446-2200) | 0,022 | 0,005 | 0.137  0.463 | 0.161  0.387 |
| TRAIL | 61  (53-68) | 47  (35-74) | 48  (44-52) | 0,41 | 0,11 | 34  (19-42) | 64  (55-75) | 0,016 | 0,864 | 0.233  0.208 | 0.275  0.134 |
| VEGF-α | 180  (130-310) | 343  (291-382) | 437  (305-557) | 0,23 | 0,11 | 504  (265-729) | 467  (312-519) | 0,056 | 0,181 | -0.012  0.948 | 0.110  0.557 |
| Cytokines, chemokines and decoy receptors selected for their relevance in anti-viral immune response or showing significant changes within patient group or between patient groups and healthy subjects. Values expressed as medians (interquartile range). HCs= Healthy controls; ICU= Intensive Care Unit. ^a^ Comparisons performed using Mann-Whitney U test. ^b^ Analysis performed using linear correlations. | | | | | | | | | | | |
